# Protein Kinase C Phosphomimetics Alter Thin Filament Ca^2+^ Binding Properties

**DOI:** 10.1371/journal.pone.0086279

**Published:** 2014-01-22

**Authors:** Bin Liu, Joseph J. Lopez, Brandon J. Biesiadecki, Jonathan P. Davis

**Affiliations:** Department of Physiology and Cell Biology, The Ohio State University, Columbus, Ohio, United States of America; Loyola University Chicago, United States of America

## Abstract

Adrenergic stimulation modulates cardiac function by altering the phosphorylation status of several cardiac proteins. The Troponin complex, which is the Ca^2+^ sensor for cardiac contraction, is a hot spot for adrenergic phosphorylation. While the effect of β-adrenergic related PKA phosphorylation of troponin I at Ser23/24 is well established, the effects of α-adrenergic induced PKC phosphorylation on multiple sites of TnI (Ser43/45, Thr144) and TnT (Thr194, Ser198, Thr203 and Thr284) are much less clear. By utilizing an IAANS labeled fluorescent troponin C, 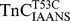, we systematically examined the site specific effects of PKC phosphomimetic mutants of TnI and TnT on TnC’s Ca^2+^ binding properties in the Tn complex and reconstituted thin filament. The majority of the phosphomemetics had little effect on the Ca^2+^ binding properties of the isolated Tn complex. However, when incorporated into the thin filament, the phosphomimetics typically altered thin filament Ca^2+^ sensitivity in a way consistent with their respective effects on Ca^2+^ sensitivity of skinned muscle preparations. The altered Ca^2+^ sensitivity could be generally explained by a change in Ca^2+^ dissociation rates. Within TnI, phosphomimetic Asp and Glu did not always behave similar, nor were Ala mutations (used to mimic non-phosphorylatable states) benign to Ca^2+^ binding. Our results suggest that Troponin may act as a hub on the thin filament, sensing physiological stimuli to modulate the contractile performance of the heart.

## Introduction

The heart is a highly dynamic organ that can regulate both its contractile strength and speed to accommodate the demands of the body [1]. A critical way to regulate cardiac function is through adrenergic pathways. For instance, enhanced cardiac function occurs during the “fight or flight response” due to increased β-adrenergic-tone. It is well established that altered intracellular Ca^2+^ signaling contributes to the altered cardiac performance upon adrenergic stimulation [2], [3]. On the other hand, adrenergic stimulation and its subsequent phosphorylation of contractile proteins also alters how the heart responds to the Ca^2+^ signal [4]. Numerous studies have demonstrated that the PKA-dependent TnI phosphorylation at Ser23/24 desensitizes the myofilament to Ca^2+^, accelerates thin filament deactivation and contributes to faster relaxation of the heart [5]. Thus, adrenergic stimulation regulates cardiac function through altering both intracellular Ca^2+^ signaling and myofilament responsiveness to the Ca^2+^ signal.

Troponin (Tn) is the Ca^2+^ sensor in cardiac muscle responsible for translating the intracellular Ca^2+^ signal into mechanical force [6]. The Ca^2+^ sensitivity of Tn can be modulated by multiple factors, including its interactions with other myofilament proteins, cardiac disease-related protein modifications as well as post-translational modifications of myofilament proteins, such as those that occur during adrenergic stimulation [7], [8], [9], [10]. In this regard, Tn is not just a passive element that transmits the Ca^2+^ signal. Instead, it may act as a central hub that integrates information from the myofilament (physiological and patho-physiological) to adjust its Ca^2+^ binding properties and regulate cardiac muscle mechanics [4].

TnC, troponin I (TnI), and troponin T (TnT) form the trimeric Tn complex that regulates myofilament activation. Both TnI and TnT are important targets of adrenergic stimulation induced phosphorylation [5], [11]. While the effect of β-adrenergic related PKA phosphorylation of troponin I at Ser23/24 is well established, the effects of α-adrenergic induced PKC activation and subsequent phosphorylation at multiple sites on TnI (Ser43/45 and Thr144) and TnT (Thr194, Ser198, Thr203 and Thr284) is much less clear. This is mainly due to the fact that several PKC isozymes phosphorylate TnI and TnT at multiple sites with different specificity, time course and extent [5], [12], [13]. Additionally, some of the PKC ioszymes can also phosphorylate the canonical PKA sites within TnI (Ser23/24) further confounding the effects of PKC phosphorylation of Tn [5], [14]. The mixed level of phosphorylation at several sites makes it difficult to dissect the site specific effects.

In this study, we generated a series of phosphomimetic mutants of TnI and TnT at multiple PKC phosphorylation sites to systematically examine the site-specific effects on Tn’s Ca^2+^ binding properties. We studied not only the effects of these phosphomimetic mutants on Tn’s Ca^2+^ sensitivity, but also their effects on Tn’s Ca^2+^ exchange kinetics, which may be even more significant to how the heart performs since the heart is dynamic and does not function in a static steady-state. Additionally, we examined the effects of different substitution residues that mimic different phosphorylation states (Asp or Glu for phosphorylation and Ala for non-phosphorylatable) on Tn’s Ca^2+^ binding at select sites. Our results show that all the phosphorylation related protein modifications of TnI or TnT alter Tn’s Ca^2+^ binding in a way that can be related to previous physiological studies. These results are consistent with the notion that Tn acts as a central hub on the thin filament by sensing physiological stimuli to alter cardiac contractile properties.

## Materials and Methods

### Materials

Phenyl-Sepharose CL-4B, Tween-20, and EGTA were purchased from Sigma Chemical Co. (St. Louis, MO). IAANS and phalloidin were purchased from Invitrogen (Carlsbad, CA). Affi-Gel 15 affinity media was purchased from Bio-Rad (Hercules, CA).

### Protein Mutagenesis

The pET3a plasmid encoding human cardiac TnC was a generous gift from Dr. Lawrence Smillie (University of Alberta, Canada). The pET3a plasmids encoding human cardiac TnI and TnT were graciously provided by Dr. James Potter (University of Miami, FL). TnC, TnI and TnT mutants were constructed from their respective pET3a plasmids using the primer-based QuikChange Site-Directed Mutagenesis Kit (Stratagene, Santa Clara, CA) as previously described [7]. The mutations were confirmed by DNA sequence analysis at an on-site molecular genetics core facility.

### Protein Purification, Fluorescent Labeling and Reconstitution of Troponin Complexes and Regulated Thin Filaments

The plasmid encoding human cardiac TnC was transformed into *E. coli* BL21(DE3)pLysS cells (Novagen, San Diego, CA), while the TnI and TnT plasmids were transformed into Rosetta™(DE3)pLysS cells (Novagen, San Diego, CA). The proteins were expressed and purified as previously described [15].

Rabbit skeletal actin and bovine ventricular tropomyosin (Tm) were purified from acetone powders as previously described [16], [17]. Fresh bovine cardiac muscle was purchased from The Herman Falter Packing Company (Columbus, OH).

TnC^C35S,T53C,C84S^ (herein denoted as TnC^T53C^) was labeled with the environmentally sensitive thiol-reactive fluorescent probe IAANS as previously described [15].

The reconstituted Tn complexes and regulated thin filaments were prepared as previously described [15].

### Steady-State Fluorescence Measurements

All steady-state fluorescence measurements were performed using a Perkin-Elmer LS55 spectrofluorimeter at 15°C. IAANS fluorescence was excited at 330 nm and monitored at 450 nm as microliter amounts of CaCl_2_ were added to 2 ml of each labeled Tn complex (0.15 µM) in a titration buffer (200 mM MOPS (to prevent pH changes upon addition of Ca^2+^), 150 mM KCl, 2 mM EGTA, 1 mM DTT, 3 mM MgCl_2_, 0.02% Tween-20, pH 7.0) with constant stirring. Reconstituted thin filaments were titrated in an identical buffer composition (excluding Tween-20). The [Ca^2+^]_free_ was calculated using the computer program EGCA02 developed by Robertson and Potter [18]. The Ca^2+^ sensitivities were reported as a dissociation constant K_d_, representing a mean of three to four separate titrations ± S.E.M. The data were fit with a logistic sigmoid function (mathematically equivalent to the Hill equation), as previously described [19].

### Stopped-Flow Fluorescent Measurements

Ca^2+^ exchange rates were characterized using an Applied Photophysics model SX.20 stopped-flow instrument with a dead time of 1.4 ms at 15°C. IAANS fluorescence was excited at 330 nm. The IAANS emission was monitored through either a 420–470 nm band-pass interference filter for 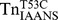, or a 510 nm broad band-pass interference filter for the thin filament. The filters were purchased from Oriel (Stratford, CT). Data traces (an average of 3 to 5 individual traces) were fit with a single exponential equation to calculate the kinetic rates. The working buffer used for the kinetic measurements was 10 mM MOPS, 150 mM KCl, 1 mM DTT, 3 mM MgCl_2_, 0.02% Tween-20 (excluded for thin filament kinetic measurements), at pH 7.0. 10 mM EGTA was utilized to remove 200 µM Ca^2+^ from the Tn complexes or thin filaments.

### Data Analysis and Statistics

Statistical significance was determined by ANOVA followed by a Dunnett’s post-hoc t-test using the statistical analysis software Minitab (State College, PA). Two means were considered to be significantly different when the P value was < 0.05. The data is shown as a mean value ± S.E.M.

## Results

### Effects of the Protein Modifications on the Ca^2+^ Sensitivity of the Tn Complex

**Figure 1:**
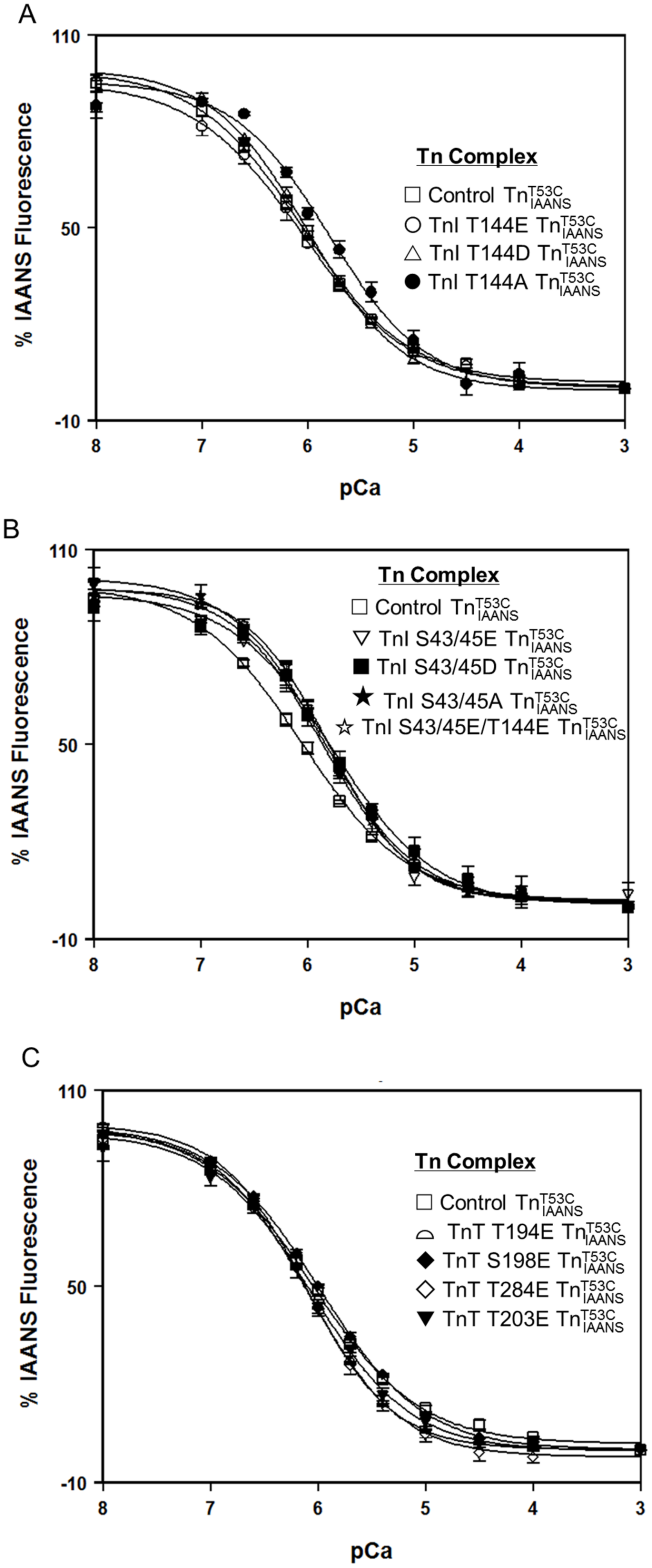
Effect of phosphorylation mimicking protein modifications on the Ca^2+^ sensitivity of the Tn complex. Panel A shows the Ca^2+^ dependent decreases in IAANS fluorescence for control 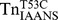 (□), TnI T144E 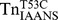 (○), TnI T144D 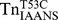 (▵) and TnI T144A 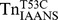 (•) as a function of pCa. Panel B shows the Ca^2+^ dependent decreases in IAANS fluorescence for control 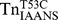 (□), TnI S43/45E 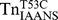 (▽), TnI S43/45D 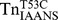 (▪), TnI S43/45A 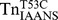 (★), and TnI S43/45E/T144E 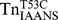 (☆) as a function of pCa. Panel C shows the Ca^2+^ dependent decreases in IAANS fluorescence for control 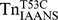 (□), TnT T194E 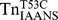 (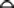), S198E 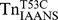 (♦), T284E 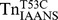 (◊) and TnT T203E 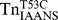 (▾) as a function of pCa. The data sets were normalized individually for each mutant.

PKC activation is associated with both increases and decreases in Ca^2+^ sensitivity of force development as well as delayed and accelerated relaxation kinetics [12], [20], [21], [22], [23], [24]. The effects of the phosphomimetic protein modifications on the Ca^2+^ binding properties of the Tn complex were examined first since Tn is the simplest biochemical system to test the effects of TnI and TnT modifications on TnC. The Ca^2+^ sensitivity of TnC within the various Tn complexes was measured by following the Ca^2+^ dependent decrease in IAANS fluorescence. Similar to previous studies [7], [15], [25], control 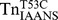 exhibited a Ca^2+^ induced half-maximal fluorescence decrease at 0.89±0.02 µM (Figure 1 and Table 1).

**Table 1:** Effect of Phosphorylation Mimicking TnI and TnT Mutants on the Ca^2+^ Binding Properties of the Tn complex.

Protein	Tn Ca^2+^ K_d_(µM)	Tn n_H_	Tn Ca^2+^ K_off_ (/s)
control	0.89±0.02	0.88±0.02	41.3±0.2
TnI T144E	0.90±0.09	0.85±0.02	40±1
TnI T144D	0.99±0.06	0.96±0.04	49±1*
TnI T144A	1.60±0.05*	0.96±0.07	50±1*
TnI S43/45E	1.4±0.1*	1.04±0.06	62±1*
TnI S43/45D	1.8±0.2*	0.91±0.04	63±1*
TnI S43/45A	1.4±0.1*	1.01±0.07	60±2*
TnI S43/45E/T144E	1.6±0.1*	1.13±0.06*	66±1*
TnT T194E	0.78±0.04	1.08±0.03*	40.6±0.4
TnT S198E	1.03±0.09	0.90±0.03	42.1±0.9
TnT T284E	0.84±0.09	1.02±0.04	40±1
TnT T203E	0.86±0.05	0.93±0.06	42.0±0.5

* significantly different from their respective control values (p<0.05).

To study the effect of PKC phosphorylation of TnI at residues Thr 144, the Thr residue was replaced by Glu, Asp or Ala individually to mimic different phosphorylation states of the site (T144E and T144D for phosphorylated while T144A for non-phosphorylatable). As shown in Fig. 1A, T144E and T144D had no effect on steady state Ca^2+^ sensitivity of the Tn complex. However, T144A significantly decreased the Ca^2+^ sensitivity of the Tn complex (∼1.8 fold, Fig. 1B and Table 1). Similarly, to study the effect of PKC phosphorylation of TnI at residues Ser43/45, the Ser residues were individually replaced by Glu, Asp, or Ala to mimic different phosphorylation states of the site. As shown in Fig. 1B, all three protein modifications decreased the Ca^2+^ sensitivity of the Tn complex, ∼1.6, ∼2 and ∼1.6 fold for TnI S43/45E, S43/45D, and S43/45A, respectively (Fig. 1B and table 1). While TnI T144E by itself had no effect on the Ca^2+^ sensitivity of the Tn complex, when combined with S43/45E, TnI S43/45E/T144E also decreased the Ca^2+^ sensitivity of the Tn complex ∼1.8 fold (Fig. 1B, Table 1). To study the effects of PKC phosphorylation of TnT, residues Thr194, Ser198, Thr203, and Thr284 of TnT were individually replaced by Glu. As shown in Fig. 1C, none of these PKC mimicking protein modifications of TnT had any effect on the steady state Ca^2+^ sensitivity of the Tn complex (Fig. 1C and Table 1).

### Effects of the Protein Modifications on the Rate of Ca^2+^ Dissociation from the Tn Complex

**Figure 2:**
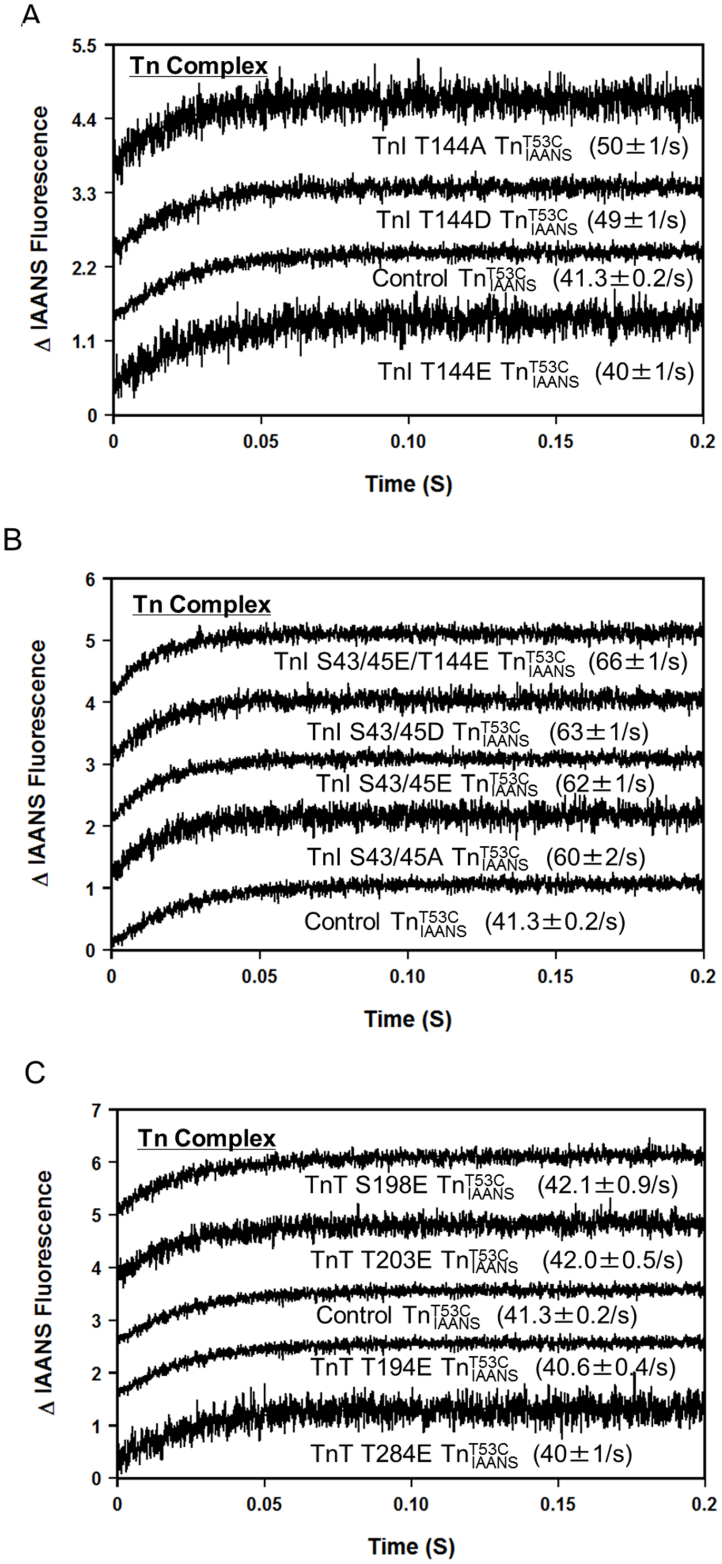
Effect of phosphorylation mimicking protein modifications on the rate of Ca^2+^ dissociation from the Tn complex. Panel A shows the time courses of the increase in IAANS fluorescence as Ca^2+^ was removed by EGTA from control 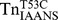, TnI T144E 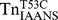, TnI T144D 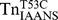 and TnI T144A 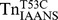. Panel B shows the time courses of the increase in IAANS fluorescence as Ca^2+^ was removed by EGTA from control 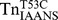, TnI S43/45E 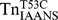, TnI S43/45D 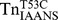, TnI S43/45A 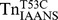, and TnI S43/45E/T144E 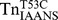. Panel C shows the time courses of the increase in IAANS fluorescence as Ca^2+^ was removed by EGTA from control 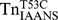, TnT T194E 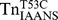, S198E 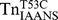, T284E 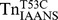 and TnT T203E 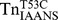. The data traces have been staggered and normalized for clarity.

Previously, we demonstrated that the fluorescence of 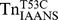 reported the actual rate of Ca^2+^ dissociation from unlabeled wild type Tn and a series of rationally engineered TnC mutants with high fidelity [7], [15]. Similar fluorescence stopped-flow measurements were performed to determine the rate of Ca^2+^ dissociation from the phosphomimetic 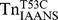 complexes. Similar to previous studies [7], [15], [25], figure 2 shows that the rate of Ca^2+^ dissociation from control 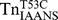 was 41.3±0.2/s (Table 1).

Consistent with its effect on the Ca^2+^ sensitivity of the Tn complex, PKC phosphorylation mimicking TnI T144E had no effect on the rate of Ca^2+^ dissociation from the Tn complex. However, TnI T144D and T144A slightly increased the rate of Ca^2+^ dissociation from the Tn complex (∼1.2 fold for both, Fig. 2A and Table 1). As shown in Fig. 2B, consistent with their effect on decreasing the Ca^2+^ sensitivity of the Tn complexes, PKC phosphorylation mimicking TnI S43/45E, S43/45D, S43/45A and S43/45E/T144E all similarly increased the rate of Ca^2+^ dissociation from the Tn complexes (∼1.5 fold, Fig. 2B and Table 1). As shown in Fig. 2C, consistent with their lack of effect on steady state Ca^2+^ sensitivity of the Tn complexes, TnT phosphomimetics (T194E, S198E, T203E, and T284E) did not alter the rate of Ca^2+^ dissociation from the Tn complex (Fig. 2C and Table 1).

### Effects of the Protein Modifications on the Ca^2+^ Sensitivity of the Thin Filament

**Figure 3:**
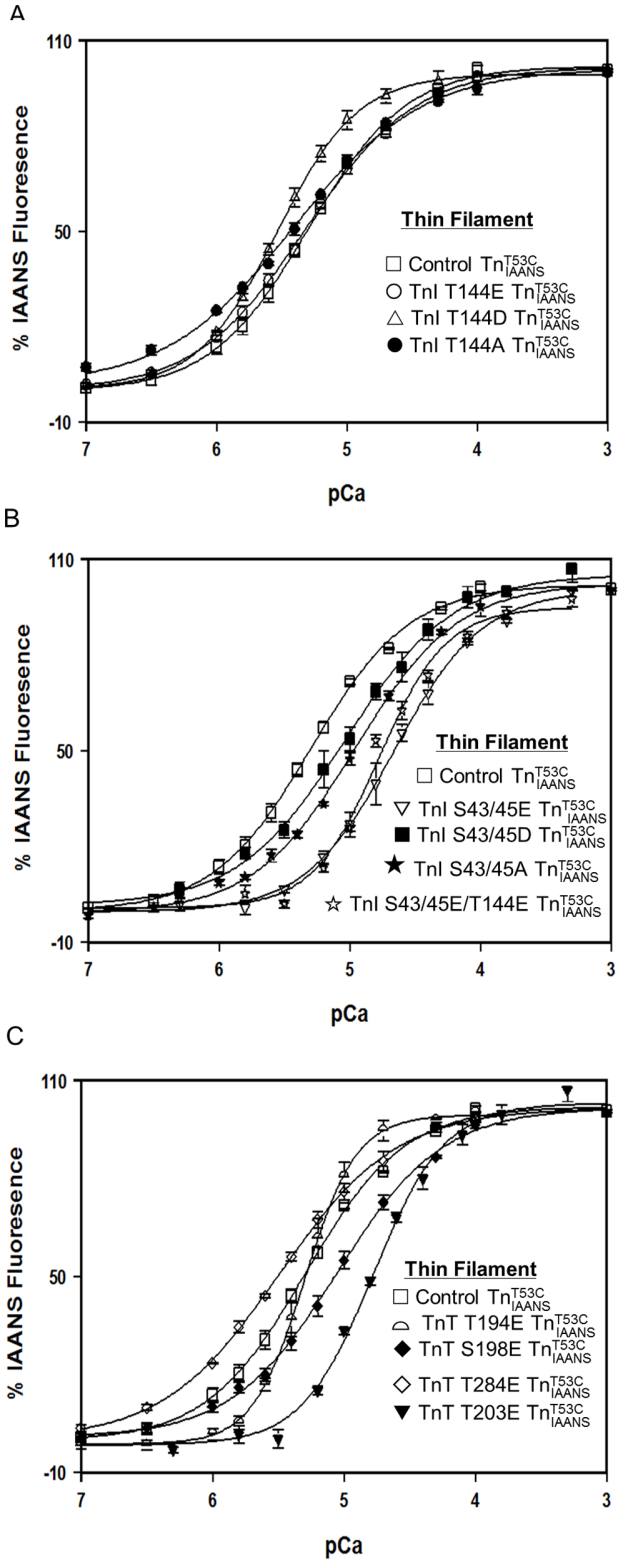
Effect of phosphorylation mimicking protein modifications on the Ca^2+^ sensitivity of the thin filament. Panel A shows the Ca^2+^ dependent increases in IAANS fluorescence for control 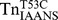 (□), TnI T144E 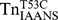 (○), TnI T144D 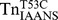 (▵) and TnI T144A 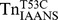 (•) as a function of pCa. Panel B shows the Ca^2+^ dependent increases in IAANS fluorescence for control 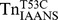 (□), TnI S43/45E 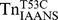 (▽), TnI S43/45D 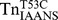 (▪), TnI S43/45A 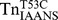 (★), and TnI S43/45E/T144E 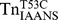 (☆) as a function of pCa. Panel C shows the Ca^2+^ dependent increases in IAANS fluorescence for control 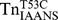 (□), TnT T194E 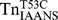 (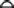), S198E 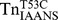 (♦), T284E 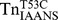 (◊) and TnT T203E 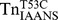 (▾) as a function of pCa. The data sets were normalized individually for each mutant.

Tn is part of the thin filament system. Previous studies have suggested that the reconstituted thin filament is the minimal physiologically relevant biochemical model system [15], [25], [26]. Accordingly, we further examined the effects of phosphomimetic TnI and TnT modifications on the Ca^2+^ binding properties of the reconstituted thin filament. By following the Ca^2+^ dependent increase in IAANS fluorescence, thin filament bound control 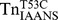 exhibited a Ca^2+^-dependent half-maximal fluorescence increase at 5.0±0.3 µM (Figure 3 and Table 2). Thus, actin-Tm decreases the apparent Ca^2+^ sensitivity of the Tn comlex.

**Table 2:** Effect of Phosphorylation Mimicking TnI and TnT Mutants on the Ca^2+^ Binding Properties of Thin Filament.

Protein	TF Ca^2+^ K_d_(mM)	RelativeChange in K_d_	TF n_H_	TF Ca^2+^ K_off_ (/s)	Relative Changein k_off_	Calculated TFCa^2+^ K_on_ (X10^6^ M^−1^s^−1^)
control	5.0±0.3	–	1.17±0.09	107.5±0.8	–	22±1
TnI T144E	4.8±0.3	1.04±0.09	1.03±0.02	101.8±0.9	1.06±0.01	21±1
TnI T144D	3.0±0.2*	↑ 1.7±0.2	1.41±0.07	76.7±0.5*	↓ 1.40±0.01	26±2
TnI T144A	3.9±0.2	↑ 1.3±0.1	0.89±0.03	105±2	1.02±0.02	27±1
TnI S43/45E	22±3*	↓ 4.4±0.7	1.31±0.01	621±24*	↑ 5.8±0.2	28±4
TnI S43/45D	9.42±0.08*	↓ 1.9±0.1	1.1±0.2	361±12*	↑ 3.4±0.1	38±1
TnI S43/45A	11±0*	↓ 2.2±0.1	1.11±0.01	208±3*	↑ 1.93±0.03	18.9±0.3
TnI S43/45E/T144E	17±1*	↓ 3.4±0.3	1.9±0.2*	509±23*	↑ 4.7±0.2	30±2
TnT T194E	5.1±0.6	1.0±0.1	2.2±0.2*	119.6±0.7	1.11±0.01	23±3
TnT S198E	9±1*	↓ 1.8±0.2	1.10±0.04	121.3±0.6	1.13±0.01	13±1
TnT T284E	3.3±0.2*	↑ 1.5±0.1	0.98±0.07	102.8±0.6	1.05±0.01	31±2
TnT T203E	17±0*	↓ 3.4±0.2	1.6±0.1	381±15*	↑ 3.5±0.1	22.4±0.9

* significantly different from their respective control values (p<0.05).

As shown in Fig. 3A, consistent with its lack of effect on Ca^2+^ sensitivity of skinned cardiac muscle force generation [24], PKC phosphorylation mimicking TnI T144E had little effect on thin filament Ca^2+^ sensitivity (Fig. 3A and Table 2). However, TnI T144D significantly increased thin filament Ca^2+^ sensitivity ∼1.7 fold (Fig. 3A and Table 2). On the other hand, the non-phosphorylation mimicking mutation, TnI T144A had little effect on thin filament Ca^2+^ sensitivity (Fig. 3A and Table 2). As shown in Fig. 3B, consistent with its Ca^2+^ desensitizing effect on skinned cardiac muscle force generation [24], PKC phosphorylation mimicking TnI S43/45E decreased thin filament Ca^2+^ sensitivity ∼4.4 fold (Fig. 3B and Table 2). Compared with TnI S43/45E, TnI S43/45D had a much smaller Ca^2+^ desensitizing effect (∼1.9 fold decrease compared with control (Fig. 3B and Table 2)). Interestingly, TnI S43/45A also decreased thin filament Ca^2+^ sensitivity ∼2.2 fold (Fig. 3B and Table 2). Additionally, when combined with T144E, TnI S43/45E/T144E decreased thin filament Ca^2+^ sensitivity ∼3.4 fold and significantly increased the cooperativity of thin filament Ca^2+^ binding (Fig. 3B and Table 2). As shown in Fig. 3C, consistent with its Ca^2+^ desensitizing effect on skinned cardiac muscle force generation [23], PKC phosphorylation mimicking TnT T203E decreased thin filament Ca^2+^ sensitivity ∼3.3 fold (Fig. 3C and Table 2). On the other hand, the mutation TnT T284E slightly increased thin filament Ca^2+^ sensitivity ∼1.5 fold (Fig. 3C and Table 2). TnT S198E decreased thin filament Ca^2+^ sensitivity ∼1.8 fold (Fig. 3C and Table 2), while TnT T194E did not alter the thin filament Ca^2+^ sensitivity, but significantly increased cooperativity of thin filament Ca^2+^ binding (Fig. 3C and Table 2).

### Effect of the Protein Modifications on the Rate of Ca^2+^ Dissociation from the Thin Filament

**Figure 4:**
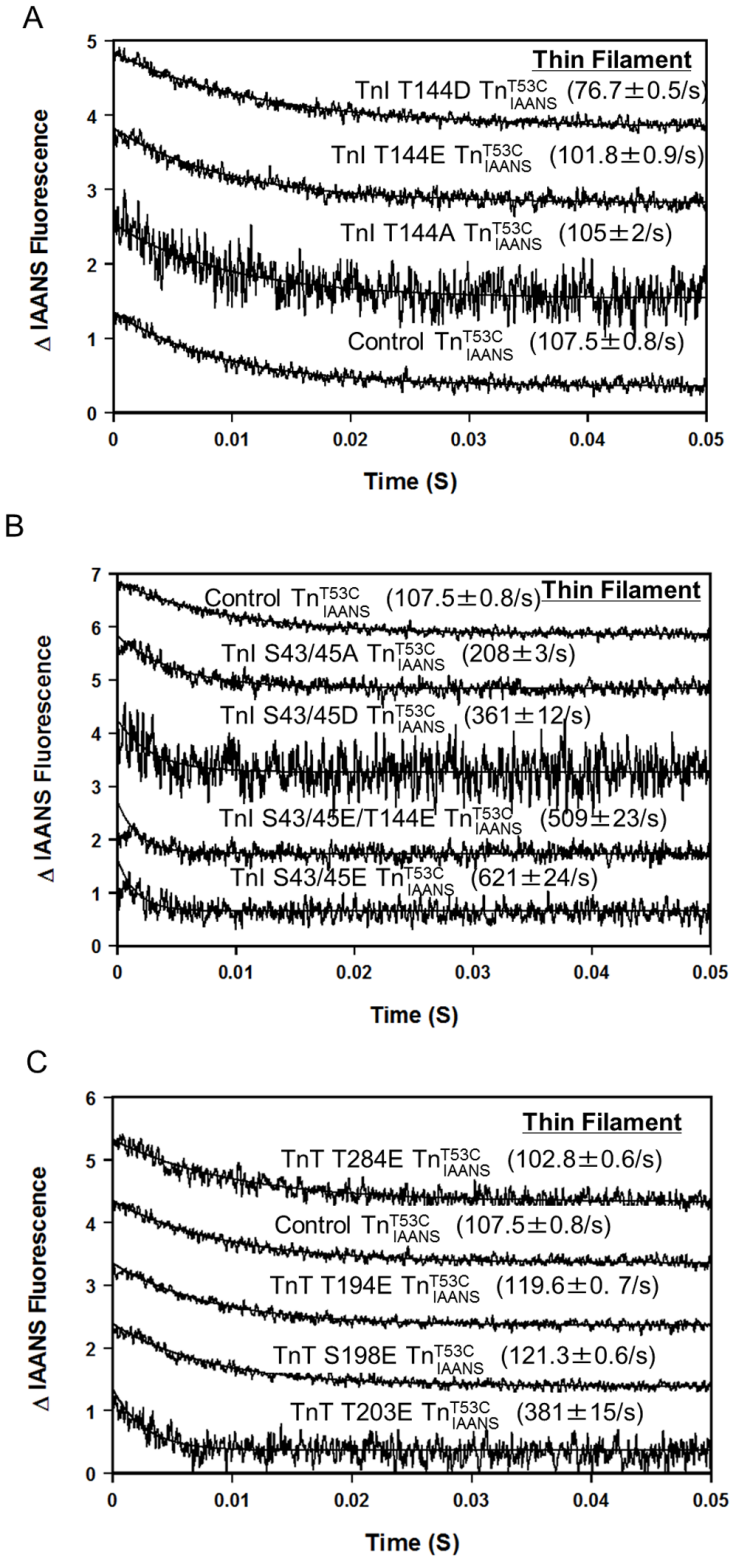
Effect of phosphorylation mimicking protein modifications on the rate of Ca^2+^ dissociation from the thin filament. Panel A shows the time courses of the decrease in IAANS fluorescence as Ca^2+^ was removed by EGTA from control 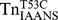, TnI T144E 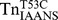, TnI T144D 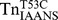 and TnI T144A 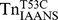. Panel B shows the time courses of the decrease in IAANS fluorescence as Ca^2+^ was removed by EGTA from control 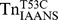, TnI S43/45E 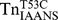, TnI S43/45D 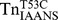, TnI S43/45A 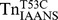, and TnI S43/45E/T144E 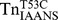. Panel C shows the time courses of the decrease in IAANS fluorescence as Ca^2+^ was removed by EGTA from control 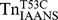, TnT T194E 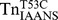, S198E 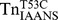, T284E 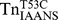 and TnT T203E 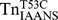. The data traces have been staggered and normalized for clarity.

Fluorescence stopped-flow measurements were conducted to determine the rate of Ca^2+^ dissociation from thin filament bound 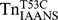 complexes. Consistent with our previous studies [25], [26], Fig. 4 shows that the rate of Ca^2+^ dissociation from thin filaments reconstituted with control 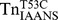 was at 107.5±0.8/s (Fig. 4 and Table 2).

As shown in Fig. 4A, consistent with its largest Ca^2+^ sensitizing effect on steady state Ca^2+^ sensitivity among the three mutations (Table 2), PKC phosphorylation mimicking TnI T144D slowed the rate of Ca^2+^ dissociation from thin filaments ∼1.4 fold (Fig. 4A and Table 2) while TnI T144E and T144A had little effect on the rate of Ca^2+^ dissociation from thin filaments (Fig. 4A and Table 2). As shown in Fig. 4B, consistent with their Ca^2+^ desensitizing effect on steady state Ca^2+^ sensitivity, PKC phosphorylation mimicking TnI S43/45E, S43/45D and S43/45A all accelerated the rate of Ca^2+^ dissociation from thin filaments (∼5.8 fold, ∼3.4 fold and ∼1.9 fold, respectively) (Fig. 4B and Table 2). Additionally, when combined with TnI T144E, TnI S43/45E/T144E increased the rate of Ca^2+^ dissociation from thin filament ∼4.7 fold (Fig. 4B and Table 2). As shown in Fig. 4C, consistent with its effect on desensitizing steady state thin filament Ca^2+^ binding, PKC phosphomimetic TnT T203E accelerated the rate of Ca^2+^ dissociation from the thin filament ∼3.7 fold (Fig. 4C and Table 2). The other three TnT mutants (TnT T194E, TnT S198E and TnT T284E) only had marginal effects on the rate of Ca^2+^ dissociation from thin filaments (Fig. 4C and Table 2).

The steady-state Ca^2+^ sensitivity of TnC is determined by the kinetics of Ca^2+^ association and dissociation. Based on the experimentally measured steady-state Ca^2+^ sensitivity and Ca^2+^ dissociation rates, we calculated the rates of Ca^2+^ association to thin filaments for each of the phosphomimetic mutants (Table 2). The majority of the phosphomimetic mutants did not alter the rate of Ca^2+^ association to thin filament.

## Discussion

In this study, we examined the effects of phosphomimetic mutants of TnI and TnT on the Ca^2+^ binding properties of TnC in the Tn complex and on the thin filament. The studied phosphorylation sites include PKC phosphorylation sites TnI Thr144, Ser43/45, and PKC phosphorylation sites TnT Thr194, Ser198, Thr203, and Thr284. Our results are consistent with physiological studies demonstrating that PKC activation can be associated with either increased or decreased Ca^2+^ sensitivity, as well as delayed or accelerated relaxation.

The majority of the phosphomimetics of TnI and TnT did not alter the Ca^2+^ binding of Tn. This is generally consistent with previous studies on the effects of familial cardiomyopathy mutants, most of which altered thin filament Ca^2+^ binding while few affected Tn Ca^2+^ binding [8], [9], [25], [27]. The exception here is the PKC phosphorylation sites TnI Ser43/45. When TnI Ser43/45 were replaced with Glu, Asp, or Ala, there were similar Ca^2+^ desensitizing effects on Tn’s steady state Ca^2+^ binding and increased kinetics of Ca^2+^ dissociation. Additionally, despite the lack of effect of TnI T144E on Tn’s Ca^2+^ binding, TnI T144D and T144A slightly affected Tn’s Ca^2+^ binding.

On the thin filament, the phosphorylation mimicking mutants altered TnC’s steady state Ca^2+^ binding in a way consistent with previous studies with skinned cardiac muscle. In a previous study on mice [24], skinned cardiac muscle was reconstituted with PKC treated TnI or phosphorylation mimicking mutants TnI S43/45E, T144E or S43/45E/T144E to examine their individual effect. Both TnI S43/45E and TnI S43/45E/T144E desensitized skinned muscle force generation to Ca^2+^, similar to PKC treated TnI, while TnI T144E had no effect [24]. Consistent with this study, our thin filament Ca^2+^ binding studies demonstrated that TnI S43/45E and TnI S43/45E/T144E desensitized thin filament Ca^2+^ binding, while TnI T144E had no effect on altering thin filament Ca^2+^ binding sensitivity. Of note, although TnI T144E had little effect on thin filament Ca^2+^ sensitivity, the other phosphomimetic mutant TnI T144D significantly increased thin filament Ca^2+^ sensitivity; this is consistent with another study which reported Thr144 was related to a Ca^2+^ sensitizing effect by PKC-βII in myocytes [12]. In an additional study on TnT phosphorylation using skinned cardiac muscle preparations, exclusive phosphorylation of TnT Thr206 (mouse sequence) by PKC and the mutant mimicking PKC phosphorylation TnT T206E depressed maximal force, ATPase activity, and myofilament Ca^2+^ sensitivity [23]. Consistent with these effects, TnT T203E (human counterpart for mouse T206E) desensitized thin filament Ca^2+^ binding in our studies. Thus, results obtained here further support that the reconstituted thin filament is a physiologically relevant biochemical system.

The thin filament kinetic studies are generally in line with the steady state studies. While the physiological significance of the Ca^2+^ dissociation rate from TnC remains controversial [4], it is striking that Tn modifications (disease or engineered) with slowed or accelerated Ca^2+^ dissociation rates prolonged or abbreviated relaxation [20], [21], [22]. PKC phosphomimetic mutants, TnI S43/45E and TnT T203E both accelerated the rate of Ca^2+^ dissociation from the thin filaments. Of note, although TnI T144E had little effect on the rate of Ca^2+^ dissociation from TnC, TnT T144D significantly slowed the rate of Ca^2+^ dissociation. Based on the extensive previous studies on PKC pathway regulation, it is highly possible that time dependent acute or chronic phosphorylation of TnI or TnT by PKC at different sites have various effects on regulating Ca^2+^ sensitivity and relaxation. Consistent with this notion, our kinetic data show that PKC phosphomimetic mutants could either accelerate (TnI S43/45E and TnT T203E) or potentially slow the rate of Ca^2+^ dissociation (TnI T144D), which may contribute to the observed acceleration and slowing of relaxation during dynamic regulation of PKC pathways [28], [29], [30].

The steady-state Ca^2+^ sensitivity of TnC is determined by the kinetics of Ca^2+^ association and dissociation. It is generally assumed that alterations in the steady-state Ca^2+^ sensitivity of TnC operate exclusively through changes in the rate of Ca^2+^ dissociation, since Ca^2+^ association to TnC has been traditionally thought to be diffusion controlled (for review see [4]). Our data clearly indicate that the majority of the phosphomimetic mutants altered the thin filament Ca^2+^ sensitivity through altering the rate of Ca^2+^ dissociation, since the magnitude of the change in the Ca^2+^ dissociation rates usually correlate well with the magnitude of the Ca^2+^ sensitivity changes (Table 2). However, TnI S43/45D and TnT S198E appear to alter Ca^2+^ sensitivity through altering both Ca^2+^ dissociation and Ca^2+^ association rates. As shown in table 2, they moderately altered the calculated Ca^2+^ association rates. These results are consistent with our previous findings that the apparent rates of Ca^2+^ association were altered by some of the disease-related protein modifications, as well as natural and engineered TnC mutations [25], [31], [32].

The next logical step to these studies is to delve into how the various PKC sites may work together to tune the Ca^2+^ binding properties of the thin filament. It will also be important to understand their combinatorial effects with the more prevalent PKA sites. We have recently demonstrated that different phosphorylation events within TnI alone (PKA and AMP activated protein kinase) cross-talk to alter the Ca^2+^ sensitivity of the thin filament [33]. It may be that the various phosphorylation sites within Tn act simply additively or combine to produce new and exciting effects on the Ca^2+^ binding properties of the thin filament.

Within the Tn literature, both Asp and Glu have been utilized to mimic phosphporylated residues [12], [24]. The validity of using one or the other amino acid substitution to mimic different phosphorylation states is not clear, thus we examined both their respective effects on TnI Ser43/45 and Thr144. Ideally the actual phosphorylated residues should be studied. However, we are unaware of experimental conditions that would allow the phosphorylation of a single site by the PKC isozymes. Both Asp and Glu are negatively charged with only slight side chain difference, while Ala is uncharged. When Thr144 was replaced by Glu (E), Asp (D), or Ala (A) to mimic different phosphorylation states, T144D was the only mutation that increased thin filament Ca^2+^ binding sensitivity and slowed the rate of Ca^2+^ dissociation. Thus, slight side chain differences between Glu and Asp could potentially cause significant differences. On the other hand, when Ser43/45 were replaced by Glu, Asp, or Ala, all three mutants desensitized thin filament Ca^2+^ binding, with the biggest effect seen for S43/45E. Interestingly, S43/45A had a bigger Ca^2+^ desensitizing effect than S43/45D, which might question the validity of trusting the Ala substitution for mimicking a non-phosphorylatable state. These results are consistent with a recent report that an Ala mutation in polo-like kinase 1 behaved similarly to both Asp and Glu pseudo-phosphorylation mutations [34].

There are potentially multiple mechanisms that exist to alter the Ca^2+^ binding properties of TnC, with the largest influence being the binding of TnI to TnC [4], [7]. Mechanisms that enhance the ability of TnI to bind the regulatory domain of TnC will increase the apparent Ca^2+^ sensitivity, whereas just the opposite will occur when TnI binding is hindered in any way [4], [7]. Considering the majority of the PKC phosphomimetics decreased the apparent Ca^2+^ sensitivity of TnC, we speculate that this is due to a decreased ability of TnC to bind TnI. Since the majority of the mutations had little effect on the isolated Tn complex, we do not think there are any major deficits in the intrinsic ability of TnC to bind TnI, but that TnI is in some way restrained from binding to TnC. This could occur more readily on the thin filament where enhanced actin-Tm binding of TnI can more effectively outcompete the binding of TnI to TnC. Thus, we predict that PKC phosphorylation of TnI and TnT increases the affinity of TnI for actin-Tm. Alternatively, phosphorylation may affect the flexibility of Tn, which can alter both the apparent Ca^2+^ sensitivity and cooperativity of the thin filament [35].

In conclusion, the phosphomimetics studied in this work altered the steady state and kinetic Ca^2+^ binding properties of TnC in a way consistent with their previously reported effects in skinned cardiac muscle or myocyte preparations. Therefore, TnC may act as a central hub that converges these physiological stimuli to affect cardiac contractile properties.
